# Cenobamate in pediatric Dravet syndrome: two responder cases highlighting the limits of simple “sodium-channel blocker” labeling

**DOI:** 10.3389/fphar.2026.1809113

**Published:** 2026-06-10

**Authors:** Konstantin L. Makridis, Linda C. Laux, Angela M. Kaindl

**Affiliations:** 1 Department of Pediatric Neurology, Charité – Universitätsmedizin Berlin, Berlin, Germany; 2 Center for Chronically Sick Children, Charité – Universitätsmedizin Berlin, Berlin, Germany; 3 Institute of Cell- and Neurobiology, Charité – Universitätsmedizin Berlin, Berlin, Germany; 4 German Center for Child and Adolescent Health (DZKJ), Section CNS Development and Neurologic Disease, Berlin, Germany; 5 Division of Neurology, Ann and Robert H. Lurie Children’s Hospital of Chicago, Chicago, IL, United States

**Keywords:** anti-seizure medication, cenobamate, developmental and epileptic encephalopathy, Dravet syndrom, epilepsy

## Abstract

Dravet syndrome (DS), caused by loss-of-function variants in *SCN1A*, is classically aggravated by chronic sodium-channel blocker treatment, reflecting impaired interneuron excitability as a core disease mechanism. Cenobamate (CNB) challenges this: adult DS cases with substantial seizure reduction have been reported, while pediatric experience is mixed and includes a small cohort with no responders and frequent worsening. We report a 7-year-old girl with *SCN1A*-associated DS and severe developmental impairment who showed marked reduction of generalized tonic–clonic seizures (GTCS) and total seizure burden with adjunctive CNB therapy. At maintenance CNB dose, which was added to valproate, clobazam and cannabidiol, a total seizure reduction of 80% was noted. An attempt to taper CNB failed because of marked seizure increase nearly back to the pre-CNB state. When CNB was returned to stable maintenance therapy levels, seizures markedly improved with no GTCS noted since October 2025. In addition, we describe a 17-year adolescent male with DS who had GTCS and daily eyelid myoclonia. He achieved suppression of seizures on low-dose CNB (50 mg/day), with only one breakthrough GTCS secondary to a missed evening medication and no reported adverse effects. Taken together with prior pediatric reports, these cases highlight the need for controlled studies to balance potential efficacy against the risk of seizure worsening when considering cenobamate in pediatric DS.

## Highlights

- Cenobamate was associated with a marked reduction in overall seizure burden in a child with *SCN1A*-associated Dravet syndrome, with the most pronounced effect on generalized tonic–clonic seizures.
- Tapering of cenobamate resulted in rapid seizure rebound, including convulsive seizures exceeding baseline frequency, while dose restoration was followed by absence of logged convulsive seizures, supporting a within-patient dose–response signal.
- Pediatric cenobamate data in Dravet syndrome remain heterogeneous and include reports of seizure worsening and treatment-emergent status epilepticus, arguing against routine use in children.
- If considered off-label, cenobamate should be restricted to specialist-supervised individualized trials with slow titration, interaction-aware management, and heightened vigilance during any dose reduction.

## Introduction

Dravet syndrome (DS) is an early-onset developmental and epileptic encephalopathy characterized by fever-sensitive prolonged convulsive seizures in infancy, evolution to multiple seizure types, developmental slowing, and elevated risk of premature mortality and SUDEP (Wirrell et al., 2022). The canonical disease model involves *SCN1A* loss of NaV1.1 function predominantly in inhibitory interneurons, leading to reduced interneuron sodium current, impaired inhibition, and network hyperexcitability (Yu et al., 2006). This interneuronopathy provides the mechanistic rationale for avoiding “classic” sodium-channel blockers (SCBs) in DS in consensus algorithms on chronic treatment.

CNB is a newer anti-seizure medication with unusually high seizure-freedom rates in randomized trials of focal epilepsy and real-world data, yet it also has sodium-channel–modulating properties that appear mechanistically differentiated from older SCBs (Makridis and Kaindl, 2023; Makridis et al., 2022a). A recent synthesis emphasizes preferential inhibition of persistent sodium current (I^NaP^) with relative sparing of transient sodium current (I^NaT^), coupled with positive allosteric modulation of extrasynaptic tonic GABA_A_ currents, features proposed to reduce paroxysmal depolarization shifts without necessarily worsening interneuron failure.

In adults with DS, we reported in a multicenter retrospective series sustained seizure reductions of >80% with adjunctive CNB therapy (Makridis et al., 2022b). Pediatric data, however, are mixed: a recent retrospective pediatric DS series reported lack of effectiveness and seizure worsening (including treatment-emergent status epilepticus), leading to discontinuation in all patients (Cagigal et al., 2025).

Here, we present two pediatric patients with genetically confirmed DS who showed clinically meaningful seizure improvement on adjunctive CNB. The primary case provides a quantitative, longitudinal seizure-diary analysis including a within-patient dose–response and withdrawal–rechallenge signal; the second case offers an independent adolescent responder phenotype at low CNB dose.

## Methods

This is a retrospective two-patient case series centered on a quantitative longitudinal seizure-diary analysis of a primary pediatric responder (Case 1) and a secondary adolescent responder (Case 2). Clinical variables were extracted from medical documentation and caregiver report. In Case 1, seizure outcomes were derived from a caregiver-maintained diary recorded prospectively as daily counts. To reduce *post hoc* bias, cenobamate exposure epochs were defined in advance according to clinically meaningful treatment milestones: initiation, titration, attainment of stable maintenance dose, taper attempt, and restoration of maintenance therapy. In Case 2, seizure outcomes were derived from caregiver-reported generalized tonic-clonic seizure dates and clinical documentation; eyelid myoclonia were clinically noted but were not available as a diary-quantified outcome with equivalent temporal granularity. Both caregivers provided permission for use of anonymized clinical information and diary-derived seizure data for publication.

## Results

### Case 1

The patient is a 7-year-old girl with DS due to a splice-site *SCN1A* loss-of-function variant (c.602+1G>A; NM_001165963.1). The patient shows severe global developmental delay and currently functions at an approximate developmental level of 2 years. The behavioral phenotype includes core features consistent with an autism spectrum disorder, with restricted and repetitive behaviors and rigid adherence to routines with poor tolerance of transitions. Communication is markedly impaired; she is minimally verbal with absent functional speech and prominent expressive language deficits. Nonverbal communication remains limited, with reduced use of gestures and eye contact.

The epilepsy proved highly pharmaco-resistant. Prior and/or concomitant therapies included levetiracetam, ketogenic diet (various ratios), cannabidiol, fenfluramine, valproic acid with levocarnitine supplementation, stiripentol, perampanel, vagus nerve stimulation, THC-A products, clobazam, and ultimately CNB. At CNB initiation she remained on multi-drug therapy including valproate, clobazam, and cannabidiol.

**FIGURE 1:**
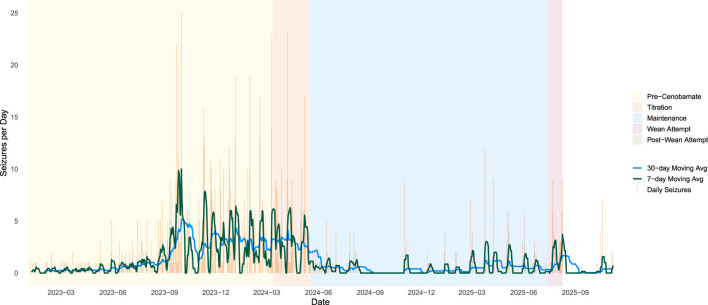
Daily seizure counts and smoothed trends across cenobamate exposure epochs. Daily seizure counts (orange bars) were extracted from the caregiver-maintained diary and plotted as seizures per day over time. Smoothed trajectories are shown as 7-day (green) and 30-day (blue) moving averages. Shaded background regions denote prespecified treatment epochs: 12-month pre-cenobamate baseline, cenobamate titration, maintenance, taper/withdrawal attempt, and post–dose restoration (post-wean attempt).

Seizure counts were prospectively recorded in a caregiver diary and aggregated into prespecified CNB exposure phases. In the 12 months preceding CNB, the overall seizure burden averaged ∼60 seizures/month (721 total; Figure 1). The cenobamate titration followed a gradual escalation to a maintenance dose of 75 mg/day (2.58 mg/kg/day), while the background regimen otherwise remained clinically stable as per caregiver documentation. During titration, seizure frequency transiently increased to ∼81 seizures/month (179 total; 3/12–5/17/24). After reaching the maintenance dose, total seizure frequency declined markedly and durably to ∼12 seizures/month (167 total over ∼14 months), corresponding to an ∼80% reduction relative to the pre-CNB baseline. A subsequent taper and withdrawal attempt, performed to enable potential inclusion in the STOKE-EMPEROR Phase 3 trial of zorevunsersen (in which cenobamate use is an exclusion criterion), was followed by a prompt rebound to approximately 56 seizures per month (7/14–8/9/25). After resumption of full-dose therapy, no seizures were logged during the post-wean observation window since October 2025. Breakthrough seizures occurred only during an acute respiratory syncytial virus infection followed by a bacterial pneumonia, consistent with fever/infection-related seizure susceptibility in DS.

Generalized tonic–clonic seizures (GTCS) showed the most salient change. CNB was associated with a marked reduction in GTCS frequency from ∼22/month at baseline to ∼3/month during maintenance. During tapering, GTCS rebounded to a level exceeding baseline; following reinstatement of the pre-taper CNB dose, no further GTCS were documented. In contrast, focal diary-coded seizures persisted during maintenance at lower residual frequency. We therefore interpret the quantitative response as most convincing for convulsive seizures rather than as uniform suppression of the full seizure spectrum. Regarding concomitant medication, clobazam dosing was stable across CNB initiation, and cannabidiol reduction occurred only after the patient had already entered the maintenance phase. These observations reduce, but do not eliminate, polytherapy-related confounding.

### Case 2

The second patient is a 17-year-old male adolescent with DS due to a variant resulting in a frame shift and a premature stop codon (c.374fs_insC; p.Thr1249insC). His neurodevelopmental profile is characterized by comparatively preserved verbal communication and adaptive skills with mild-to-moderate intellectual disability: he speaks clearly and communicates needs, reads at an approximately first–to-second grade level, performs double-digit addition, and is independent in dressing, feeding, mobility, and toileting, while requiring assistance with bathing and toothbrushing. His epilepsy phenotype comprises GTCS, with eyelid myoclonic clusters often preceding GTCS. In the year preceding CNB initiation (February 2023 through early February 2024), caregivers recorded 19 GTCS with escalation during the second half of 2023.

CNB was initiated because seizures were increasingly difficult to control despite polytherapy and vagus nerve stimulation. Concomitant anti-seizure medications (ASM) at CNB initiation and throughout follow-up were clobazam, valproic acid, ethosuximide, and fenfluramine. No ASM changes occurred during CNB titration or maintenance.

CNB was started using the standard titration pack (12.5 mg/day; 0.21 mg/kg/d), and was then increased to 50 mg once daily, which has remained the stable maintenance dose (0.86 mg/kg/d), after which no GTCS were recorded over the subsequent 12 months, with only one breakthrough GTCS secondary to a missed evening medication. No CNB-attributed adverse effects were reported. Because longitudinal quantification in this case was robust for generalized tonic-clonic seizures but not for eyelid myoclonia at the same level of temporal resolution, we restrict the principal efficacy claim in Case 2 to generalized tonic-clonic seizure suppression.

## Discussion

This report contributes two pediatric cenobamate “responder” phenotypes to a rapidly evolving literature on CNB in DS. Case 1 is rigorously diary-anchored and shows a marked reduction in total seizure burden across exposure epochs, with a particularly pronounced attenuation of generalized tonic–clonic seizures (GTCS). This within-patient withdrawal–rebound/re-challenge pattern in Case 1 is consistent with a treatment-related effect, but does not exclude spontaneous fluctuation or intercurrent destabilization in DS.

Pediatric CNB experience in DS is no longer “unknown”; it is heterogeneous with a harm signal. The most consequential dataset is the pediatric DS cohort by Cagigal et al., who reported six children treated off-label with CNB: no child met responder criteria, worsening occurred in approximately half, and one patient developed treatment-emergent status epilepticus after a prolonged status-free interval, prompting discontinuation in all cases after a median follow-up time of 7 months (range = 3–18, IQR = 9.5) (Cagigal et al., 2025). The discrepancy between our cases and the negative pediatric cohort of Cagigal et al. is unlikely to be explained by dose, titration, or clobazam exposure alone, as these factors were broadly comparable. Cagigal et al. also used low starting doses (6.25–12.5 mg), slowly titrated half of the cohort, and reported final doses of 25–100 mg/day. Concomitant clobazam exposure is likewise not a clear discriminator, as all six Cagigal patients and both of our patients received clobazam-containing polytherapy. Follow-up duration may contribute only partially: our primary case includes prolonged maintenance and a withdrawal–reinstatement sequence, but Cagigal et al. still observed nonresponse or worsening in children treated for up to 17–18 months. A more plausible explanation is seizure-type and endpoint heterogeneity: the clearest effect in our series concerned generalized tonic-clonic seizures, whereas Cagigal et al. evaluated a phenotypically mixed cohort using overall seizure frequency. Cenobamate may therefore reduce convulsive seizure burden in a subset of patients with Dravet syndrome. This interpretation remains cautious, because the Cagigal cohort also documented prolonged seizures, emergence of new bilateral tonic–clonic seizures, and treatment-emergent status epilepticus.

The historical avoidance of sodium-channel blockers in DS is mechanistically coherent. In *SCN1A* loss-of-function, interneuron excitability is compromised; further reduction of available transient sodium current (I^NaT^) risks amplifying inhibitory failure and worsening seizures (Yu et al., 2006). The category label “sodium-channel blocker,” however, collapses mechanistically distinct actions into a single class. CNB differs from classic fast-inactivation blockers in that it preferentially inhibits persistent sodium current (I^NaP^) with relative sparing of I^NaT^ and it enhances tonic GABAergic inhibition (Sankar et al., 2025). A plausible hypothesis is therefore that CNB can reduce principal-cell burst propensity and seizure propagation without materially worsening interneuron spike generation in a subset of patients (Sankar et al., 2025). In *Scn1a* ^+/−^ mice, GS967—an agent with prominent effects on persistent sodium current—improved survival and reduced seizures, suggesting that targeting aberrant sodium-current components and downstream channel remodeling can be beneficial even in an *SCN1A* haploinsufficiency state (Anderson et al., 2017). These findings do not establish CNB efficacy in DS, but they argue against categorical assumptions based solely on drug class.

Pharmacokinetic and pharmacodynamic interactions remain a critical confounder in DS, where polytherapy is the rule. CNB inhibits CYP2C19 and can substantially increase N-desmethylclobazam exposure, producing clinically relevant sedation and, in some cases, apparent seizure-control synergy when clobazam is reduced rather than eliminated. Accordingly, an observed “CNB response” may partly reflect an optimized benzodiazepine-metabolite milieu in clobazam co-treated patients, and future DS CNB reports should document clobazam dosing changes and tolerability trajectories explicitly.

## Limitations

This report has several limitations. It describes only two patients and is retrospective in design. In addition, the baseline antiseizure medication regimens differed between the two cases (Case 1: valproate, clobazam, cannabidiol; Case 2: valproate, clobazam, ethosuximide, fenfluramine), which complicates isolation of a cenobamate-specific treatment effect and limits interpretation of potential pharmacodynamic and pharmacokinetic drug-drug interactions. This is particularly relevant because cenobamate can substantially increase N-desmethylclobazam exposure, yet neither clobazam nor N-desmethylclobazam concentrations were measured. However, no clear sedation signal or clinically relevant benzodiazepine toxicity was documented during CNB co-treatment. Seizure ascertainment also differed by case and relied primarily on caregiver-derived data. Case 1 was based on a prospectively maintained caregiver seizure diary, whereas Case 2 relied on caregiver report and clinical documentation. Although clinically valuable, these sources remain vulnerable to subjective bias, ascertainment bias, and imperfect seizure-type classification. Further, standardized outcome definitions were not applied prospectively across both cases. Generalized tonic-clonic seizures are more readily recognized by caregivers than subtle focal, nonmotor, nocturnal, or electroclinical seizures, which may therefore have been underreported or misclassified. Accordingly, the strongest quantitative signal in this series concerns generalized tonic-clonic seizure reduction rather than the full seizure spectrum. In Case 2 in particular, eyelid myoclonia were described clinically, but not documented longitudinally with the same granularity as generalized tonic-clonic seizures, limiting confidence in seizure-type-specific conclusions. We also did not obtain synchronized EEG data to correlate electroclinical burden with reported clinical events. These limitations constrain interpretation of whether cenobamate exerted differential effects across seizure types and, consequently, restrict any mechanistic inference linking seizure-type-specific improvement to cenobamate pharmacology. Finally, although the withdrawal–rechallenge signal in Case 1 supports a treatment-related effect, it does not establish causality, because seizure fluctuations in Dravet syndrome may also be influenced by infection, fever, and temporal instability within polytherapy regimens.

In this report, the term “responder phenotype” is used descriptively to denote a patient with clinically meaningful and sustained seizure reduction on CNB without intolerable adverse effects under relatively stable background therapy; it does not imply a validated genotype-specific or biomarker-defined response class.

## Conclusion

As of now CNB cannot be positioned as a broadly applicable option in (pediatric) Dravet syndrome, because data show potential worsening. At the same time, accumulating adult experience and select pediatric successes—including the present cases—argue that a categorical prohibition may be overly crude, particularly for patients with ongoing seizures.

The only defensible stance is a highly cautious, specialist-supervised, individualized trial framework: slow titration; explicit monitoring for increased seizure frequency or new status epilepticus; proactive management of drug–drug interactions (especially clobazam and other sodium-channel agents); and extreme care with tapering given the clear rebound risk suggested by the Case 1 course.

## Data Availability

The datasets presented in this article are not readily available because of ethical and privacy restrictions. Requests to access the datasets should be directed to the corresponding author.
